# Efficacy of High-Intensity Training in Patients with Moderate to Severe Dysphagia after Glossectomy

**DOI:** 10.3390/jcm12175613

**Published:** 2023-08-28

**Authors:** Elena Pavlidou, Athanasios Kyrgidis, Konstantinos Vachtsevanos, Jannis Constantinidis, Stefanos Triaridis, Athanasia Printza

**Affiliations:** 1 1st Department of Otorhinolaryngology, School of Medicine, Faculty of Health Sciences, Aristotle University of Thessaloniki, 54124 Thessaloniki, Greece; e-pavlidou@hotmail.com (E.P.); janconst@otenet.gr (J.C.); triaridi@auth.gr (S.T.); 2 Department of Oral and Maxillofacial Surgery, School of Dentistry, Faculty of Health Sciences, Aristotle University of Thessaloniki, 54124 Thessaloniki, Greece; kyrgidis@auth.gr (A.K.); vaxtseva@dent.auth.gr (K.V.)

**Keywords:** dysphagia, glossectomy, swallowing, head and neck cancer, tongue pressure, tongue endurance, swallowing pressure, tongue cancer, dysphagia treatment, rehabilitation, EAT-10

## Abstract

Dysphagia is the main impairment arising from glossectomy for tongue cancer treatment. The study aimed to determine if an eight-week training protocol paired with accuracy tasks and swallowing exercises is effective and can improve tongue strength and swallowing in patients after tongue resection. Maximum isometric pressures, tongue endurance, swallowing pressures, mealtime duration, and oropharyngeal swallow function were studied in patients with moderate to severe dysphagia after glossectomy. Twenty-five (25) patients and thirty-one (31) healthy participants were enrolled in the study. The therapy group (TG) consisted of seventeen (17) patients who followed an 8-week treatment protocol and had multiple measurements. The follow-up control group (FUG) consisted of eight non-treated patients who had a baseline and an 8-week follow-up examination. Healthy participants served as the reference group (RF). Maximum isometric pressures, endurance, and swallowing pressures increased significantly in the TG versus the FUG. Significant improvement was documented in the TG regarding the EAT-10 questionnaire, the Penetration-Aspiration Scale scores at thickened and solid boluses, and post-swallow residues at thickened and solid boluses. The treatment protocol with tongue strength exercises combined with accuracy tasks and swallowing exercises improves the post-operative swallowing function in patients after glossectomy. Patients in the TG had more significant and quicker improvement in pressures and endurance compared to FUG.

## 1. Introduction

The presence of tongue cancer has been associated with various degrees of swallowing difficulties which may cause mild to severe dysphagia. Surgical treatment involves the removal of tongue tissue, also known as glossectomy [1]. Dysphagia that arises from partial glossectomy is characterized by reduced bolus control and manipulation in the oral cavity, which in turn influences swallow efficiency and increases the risk of aspiration [2,3,4]. It can also lead to dehydration, malnutrition, and a reduced quality of life [5]. Therefore, a rational and effective approach is required to treat dysphagia. If the patient can swallow safely, the goal is to improve mastication and swallowing. If the patient is at high risk for aspiration, he or she will continue to depend on a gastric tube and an efficient therapeutic protocol is required to treat severe dysphagia.

The tongue is the main integral organ involved in the oral and oropharyngeal phases of swallowing, and any resection of it results in the loss of pressure needed to propel the bolus from the oral cavity into the oropharynx and from the oropharynx into the esophagus [6,7]. Tongue pressure (TP) is defined as the force occurring between the anterior palate and the tongue. Anterior tongue pressures are necessary to anchor the tongue against the alveolar ridge to initiate bolus propulsion. However, posterior pressures are essential for stimulating the pharyngeal swallow response and contributing to hyoid ascent [8].

Evaluating the tongue pressure postoperatively in patients with head and neck cancer (HNC) might offer a safe, useful, quantitative tool to assess dysphagia [9]. It is also a helpful assessment as it determines patients’ remaining tongue muscle power, which indicates their remaining tongue function [10].

Establishing measures of tongue strength and endurance relative to “within tongue location” in patients after tongue resection is warranted. There are a few studies in the literature evaluating the pressures of the tongue in HNC patients [9,10,11]. However, these studies examine only one portion of the tongue. It has not yet been determined what pressures and endurance are needed by the different regions of the tongue (anterior portion–posterior portion) in patients after tongue resection.

Additionally, lingual swallowing pressures have been shown to differ at anterior and posterior tongue locations [8]. It should be noted that tongue strength in previous studies with head and neck cancer was measured as a separate task, rather than during the swallows. No studies exist that evaluate swallowing pressures (regular/effortful saliva swallows) in patients after tongue resection, and no reports that examine the associations between swallow measures, tongue strength, and swallow function following glossectomy. In addition, no study to date has examined the changes in swallowing pressures and function of swallowing over time in patients after glossectomy.

Finally, it has been found that there are significant associations between reduced tongue strength, longer meal durations, and reduced food consumption in long-term care residents [12]. However, objective measures of tongue endurance, tongue strength, and its effects on swallowing performance over time have not been established for patients after tongue resection.

Tongue strengthening exercises can improve impaired tongue strength, tongue endurance, and decreased tongue pressure. Tongue strengthening programs have shown improvement in lingual strength following isometric lingual exercise in healthy individuals [13,14], in neurologically impaired subjects [15,16,17], and head and neck malignancies [18,19,20,21]. Although the number of studies documenting positive outcomes has increased gradually, the patient sample is very heterogeneous, especially considering the subgroups of the HNC patients.

Dose-response data are lacking and there is a need to determine the frequency, length of treatment, and timing of the exercise program, particularly in patients after glossectomy, as there may be a limited temporal window in which treatment will have a beneficial effect. Swallowing intervention is beneficial in the tongue cancer population, but it remains unclear what the ‘active ingredients’ are for successful rehabilitation [2]. While the dysfunction of the swallowing mechanism after glossectomy is known, the effectiveness of the intervention to rehabilitate dysphagia has not been widely investigated.

The primary aim of this study was to determine the efficacy of an 8-week high-intensity intervention protocol followed by patients with moderate to severe dysphagia after glossectomy. Improvement of tongue pressure, tongue endurance, swallowing pressure, and their effects on swallowing function were investigated and documented over time. A second objective was to determine whether measures of mealtime function, such as mealtime duration, would change in association with improvements in tongue strength in patients after tongue resection following the treatment protocol.

## 2. Materials and Methods

### 2.1. Participants

Fifty-six (56) participants were included in the study, comprising twenty-five (25) patient participants who were divided into two comparator groups that underwent tongue resection for the treatment of oral cancer and thirty-one (31) healthy participants that comprised the healthy control comparator group. All participants signed the informed consent form after they were given the opportunity to review the informed consent document and discuss it with a trained examiner.

Inclusion criteria for the patients were age over 18 years, swallowing difficulties following glossectomy, a score on the EAT-10 (Eating Assessment Tool) questionnaire ≥ 3 [22], and ability to follow the treatment protocol with the speech-language pathologist (SLP). Exclusion criteria were prior head and neck cancer or pre-existing swallowing disorder, neurologic history, or injury that might affect the swallowing function or cognition. The patients included in the study were feeding either orally with soft boluses or non-orally through gastrostomy (PEG) due to aspirations.

The healthy participants were required to be over 18 years, with no swallowing difficulties, no cognitive disorders, and a score on the EAT 10 questionnaire < 3.

### 2.2. Design of the Study

The study included two patient participant groups and one healthy participant group. All participants (both patients and healthy) underwent a tongue function assessment that included the following measurements: maximum isometric pressure anterior (MIPa), maximum isometric pressure posterior (MIPp), endurance of the tongue anterior (ETa) and endurance of the tongue posterior (ETp), normal swallow pressure anterior (NSPa) and normal swallow pressure posterior (NSPp), forceful swallow pressure anterior (FSPa) and forceful swallow pressure posterior (FSPp).

The participant patients were randomly allocated to one of two patient groups: (1) The therapy group (TG) which included patients that followed the treatment protocol and performed the tongue assessment at baseline and at weeks 2, 4, 6, and 8; (2) The follow-up control group (FUG) that included patients that were evaluated only at the baseline and at week 8. Patients were offered the TG or FUG option after randomization, in a 2:1 ratio favoring TG. FUG patients still received more swallowing care compared to the institutional standard of care. All patients consented to their randomized treatment modality.

A third group, a reference group consisting of healthy participants, was also tested once to provide normative reference data specific to the study setting.

At baseline, patients who followed the treatment protocol were informed about the training protocol that would follow. The protocol comprised an 8-week lingual exercise program with isometric strength exercises and tongue pressure accuracy tasks using the Iowa Oral Performance Instrument (IOPI). The patients were also trained in the effortful swallow, the Mendelson maneuver, and the supraglottic swallow.

Training with the speech-language pathologist was performed at a frequency of two times per week, 45 min per session, not on consecutive days, to allow for sufficient rest periods. The nutritional feeding status and any specific oral diet type were documented at each session and recorded. Patients were instructed to perform traditional lingual exercises (motor and resistance lingual exercises) plus the swallowing maneuvers every day at home at an intensity of a minimum of two sets of 10 trials for each exercise for eight weeks. Daily home exercises were provided in writing to each patient and reviewed at the in-person sessions. Mealtime duration (MD), which is the time patients need to consume a meal at home, was recorded based on the patient’s report. It was based on three recordings, and the mean value of the three was documented as the MD. It was calculated in seconds and recorded at baseline and in the eighth week.

Swallow safety for the TG was based on the Fiberoptic Endoscopic Evaluation of Swallow (FEES), reported with the use of the 8-point Penetration-Aspiration Scale (PAS) for liquids, semisolids, and solids (PAS > 3 indicated unsafe swallow). Self-reporting of the swallowing difficulty was based on a total score of EAT-10 questionnaire ≥ 3. Swallow safety was evaluated at the baseline and week 8.

### 2.3. Maximum Isometric Pressures, Endurance Measurements, Bulb Position

The Iowa Oral Performance Instrument (IOPI Medical LLC, Redmond, WA, USA) was used to measure the variables related to tongue function. The tongue squeezes an air-filled bulb, and the amount of generated pressure is displayed on a digital device. IOPI also has a light display that indicates the pressure exerted relative to a manually set maximum value. An expert SLP explained the procedure to the participants in order to complete the tasks. Each participant was placed in a relaxed sitting position and the bulb was placed firstly on the anterior and secondly on the posterior portion of the tongue.

As the patients had lower tongue volume due to the resection, attention was paid to the position of the bulb in order to ensure that it was in a steady and comfortable placement. Once the bulb was appropriately positioned, the speech-language pathologist indicated the point where the tubing running from the intraoral bulb to the connective tube met the upper incisors using a permanent marker to allow reliable bulb placements between trials [23].

Participants were then instructed to raise the tongue and compress the bulb against the roof of the mouth as strongly as possible for 3 s. Measurements were repeated three times and the highest value of the three pressures was used as the subject’s maximum tongue strength [23]. A 2 min rest was given to the participants between the trials to avoid fatigue.

Tongue endurance was set to 50% of the MIP. Participants were asked to maintain 50% of MIP for as long as possible, and the time was recorded. The trials were terminated when the participant could no longer sustain adequate pressure to keep the lights within the green zone. Three trials of tongue endurance were performed and two minutes of rest between trials was given to each participant. The greatest value of the trials was used as the endurance for each participant [24]. Maximum isometric tongue pressure was measured in kilopascals (kPa,) and endurance in seconds (s).

### 2.4. Swallowing Pressures

Maximum tongue pressures during saliva swallows were recorded with the participant sitting in a relaxed position. Both normal and forceful swallows were dry since many patients were feeding non-orally or partially orally. For the normal swallow, the participant was instructed to swallow his/her saliva normally and the maximum tongue pressure was recorded. For the effortful swallow, subjects were instructed to swallow hard, emphasizing tongue-to-palate contact. The SLP instructed and encouraged the participant verbally (“As you swallow, push your tongue really hard against the roof of your mouth and then swallow”) [25].

Each participant performed each swallow three times and the highest pressure was recorded. In order to minimize muscle fatigue, each subject rested for more than 2 min between tasks.

### 2.5. Dysphagia Screening Test (EAT-10), FEES, Penetration-Aspiration Scale (PAS), Post-Swallow Residues (PSR)

The dysphagia screening test EAT-10 is a self-questionnaire that screens oropharyngeal dysphagia. It is a reliable, valid, and quick tool, validated in many languages [26]. Each question is scored from 0 to 4 (“no problem” to “severe problem”). The total EAT-10 score is calculated by adding the score of each question. All participants completed the EAT-10 questionnaire by themselves before performing the tasks with the IOPI. Patient self-reported outcome measures are important measurement tools for the status of the patient’s health condition and the detection of dysphagia [27,28].

Swallows captured via flexible endoscopy were evaluated using the 8-point Penetration-Aspiration Scale (PAS) to identify the presence and degree of any prandial material within the airway [29]. If a patient was unable to swallow a particular bolus due to aspiration, repeated boluses were not given. When aspiration was occurring, postures, maneuvers, or a combination of them were performed in order to eliminate it. When aspiration could not be eliminated, the FEES was terminated, and the PAS scale was defined, which ranged from 1 (material does not enter the airway) to 8 (material enters the airway, passes below the vocal folds, and no effort is made to eject).

Post-swallow residues in the valleculae, pyriform sinuses, or pharyngeal wall with either a spoon-thick or solid bolus were recorded during FEES using a 4-point ordinal scale: 0 = none, 1 = thin coating, 2 = 25–50% full, 3 = 50% full [30]. PAS scale and PSR scale were only performed in participants in the TG pre-post treatment.

### 2.6. Training Protocol

Patients in the TG performed a high-intensity 8-week treatment protocol which included isometric strength exercises and tongue pressure accuracy tasks. Tongue muscle strength training was classified into the anterior and posterior regions. Before beginning the exercise program, a baseline 1-repetition maximum (1-RM) pressure was identified. A 1-RM was defined as the highest amount (i.e., pressure) that can be generated one time [15] and is used to define the resistive load and thus the intensity of resistance training for the accuracy task [31]. A training intensity of 60–80% 1 RM has been shown to be effective in improving the strength of the skeletal muscles. Our protocol was based on Robbins et al. [15], who reports increases in tongue strength and volume in stroke patients following an 8-week resistance training protocol, performing a resistance between 60–80% 1 RM.

The resistance was set by the speech-language pathologist at a 60–80% level of maximal isometric tongue pressure. Patients were instructed to press the sensor until the level was reached and to maintain it there for 2 s. An intensity of two sets of 10 repetitions each for the anterior and posterior regions of the tongue was performed by each patient and the patients were instructed to cease the exercises if they felt discomfort or pain in the tongue during the exercises.

### 2.7. Statistical Analysis

Demographic, clinical, patient-reported, and physician-recorded variables from the registry were included in the analysis. Scale variables were checked for normality. Non-parametric tests (Mann–Whitney U-test, Kruskal–Wallis test, and Wilcoxon signed-rank test) were used where appropriate. For qualitative variables, Pearson’s chi-squared or Fisher’s exact tests were used. Nonparametric and parametric paired tests were used. Relative frequencies for demographic and clinical variables were obtained. Spearman’s q coefficient was used to flag significant correlations. The type I error probability associated with all tests in this study was set to 0.05. Statistical analyses were performed using SPSS software version 23.0 (IBM Corp., Armonk, NY, USA).

## 3. Results

### 3.1. Participants’ Characteristics

A total of 56 participants were included in the study. The TG consisted of seventeen (17) patients, the FUG group of eight (8) patients, and the RG of thirty-one (31) healthy participants. The baseline characteristics of the groups are presented in Table 1. The period between the surgical treatment and our baseline evaluation varied from 2 to 10 months, with most patients (21) having the baseline assessment in 3–4 months.

### 3.2. Maximum Isometric Pressures, Tongue Endurance, and Swallowing Pressures

Our analysis showed that overall, we had recorded improvement between baseline and week 8 in all our patients. Statistically significant improvement appeared in all tongue pressures in the TG pre-post treatment: MIPa (*p* < 0.001), MIPp (*p* < 0.001), ETa (*p* < 0.001), ETp (*p* < 0.001), NSPa (*p* < 0.001), NSPp (*p* < 0.001), FSPa (*p* < 0.001), and FSPp (*p* < 0.001).

**Figure 1:**
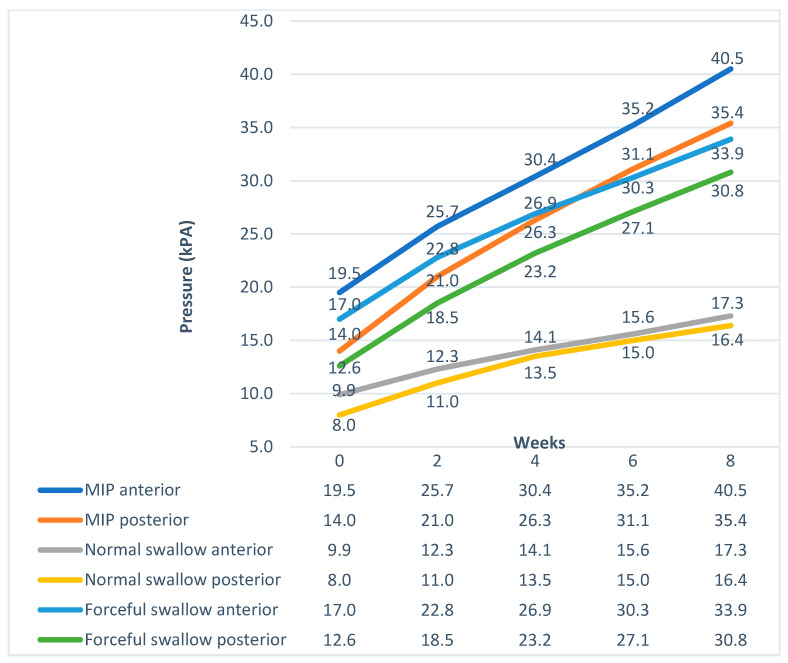
Pressure progress over time in the Therapy Group.

**Figure 2:**
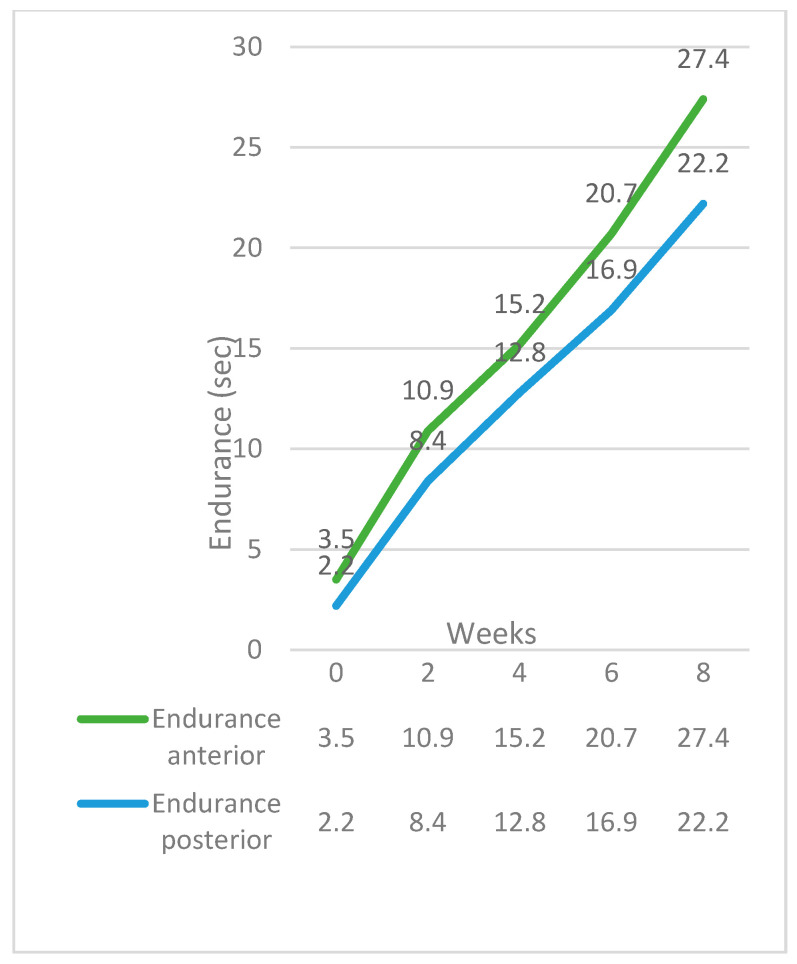
Endurance progress over time in the Therapy Group.

**Figure 3:**
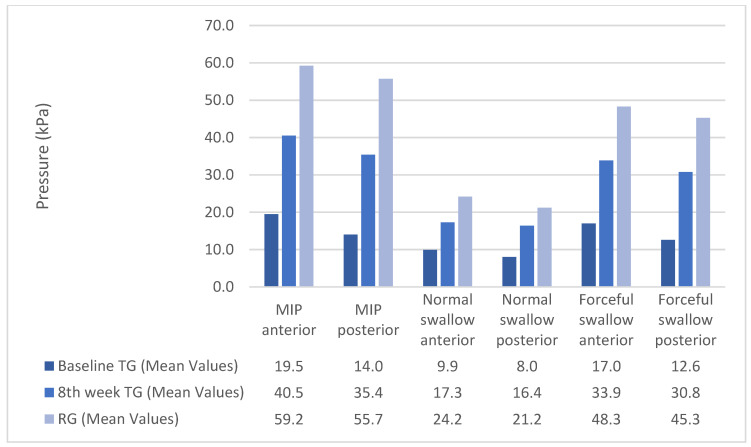
Pressure pre-post treatment in the Therapy Group versus the Reference Group.

The maximum benefit in maximum isometric pressures, swallow pressures, and endurance appeared at the end of the second week. From that point, the pressures and endurance continued to increase almost steadily until the end of the training program (Figure 1 and Figure 2). Although the pressures increased significantly for the TG, post-treatment, they were still lower than normal participants (Figure 3). Endurance of the tongue for both anterior and posterior portions in the TG post-treatment appeared to be higher compared to the CG (CG: ETa: 21.6″, CG: ETp: 17.6″).

**Figure 4:**
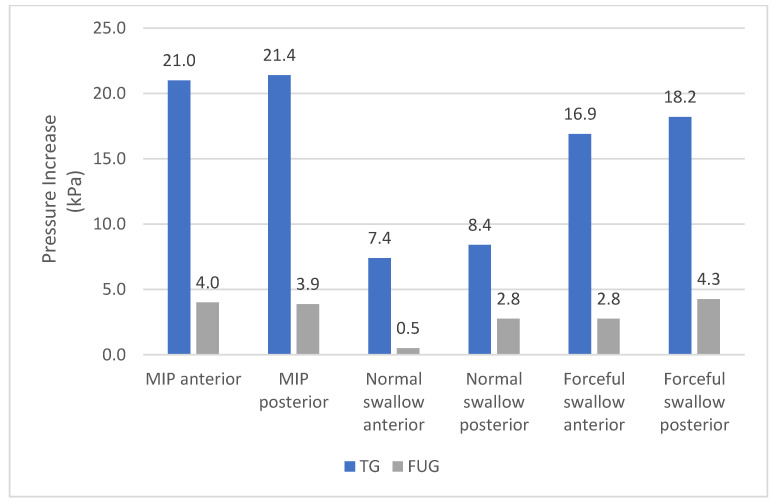
Increase of the pressure after eight weeks in the Therapy Group versus the Follow-up Group.

**Figure 5:**
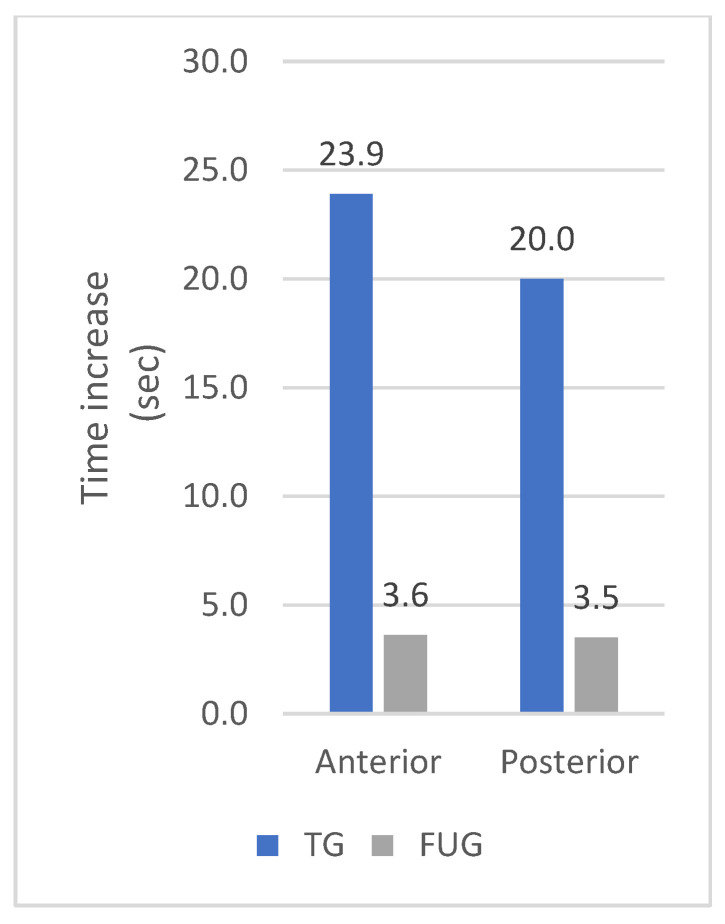
Increase of the endurance after eight weeks in the Therapy Group versus the Follow-up Group.

Patients in the FUG also showed statistically significant improvements in MIPa (*p* = 0.001), MIPp (*p* = 0.015), ETa (*p* < 0.001), and ETp (*p* = 0.002) after eight weeks compared to the baseline. Although the swallowing pressures improved in the FUG group, there was no statistically significant increase. The differences in the pressure increase and the endurance increase between the TG and the FUG are presented in Figure 4 and Figure 5.

### 3.3. Dysphagia Screening Test (EAT-10), PAS, and PSR

Swallow function and oral intake also improved in the TG. Mean values and standard deviations of the baseline scores and the improvement in the oropharyngeal measures are presented in Table 2. Mealtime duration also decreased significantly at the end of the treatment.

At the baseline, seven patients with PEG aspirated at all consistencies. Ten (10) patients were feeding orally only with soft boluses. All these patients had difficulty with thin liquids using small sips. At the end of the treatment protocol, two patients with PEG started total oral feeding and removed the PEG, and five patients with PEG started additional oral intakes. Two patients with PEG were diagnosed with cancer relapse at the end of the sixth week and started additional adjuvant treatment. Twelve (12) patients started feeding with all consistencies post-treatment. The difference between the feeding status across all time points was statistically significant (*p* < 0.001) pre-post treatment. According to the EAT-10 scale, patients in the FUG continued to have difficulties with solid boluses at the end of the 8th week. However, since the EAT-10 < 3, the PAS scale was not performed.

## 4. Discussion

### 4.1. Maximum Isometric Pressures and Swallow Exercises

Our study showed that all patients at the baseline had low pressures for both anterior and posterior tongue measurements. The study aimed to provide information regarding the lingual pressures in the anterior and particularly posterior tongue, which arguably provide critical forces necessary for safe and effective oropharyngeal swallowing [8]. We also combined strengthening and swallowing exercises for the optimal swallow outcome. Swallowing exercises have been found to improve tongue pressures [32] and thereby swallowing function. Since most of our patients had moderate to severe dysphagia and non-oral nutritional intake, the goal of the treatment protocol was to minimize the aspirations and increase swallow safety.

The TG showed greater improvement compared to the FUG and the difference between them was immense. Although the increase in both groups at baseline and in the eighth week was statistically significant, the pressure increase achieved by the FUG in eight weeks was the same achieved by the TG in approximately ten days.

Robbins et al. performed tongue-strengthening exercises in older individuals. The maximum tongue pressure was increased by approximately 6 kPa after four weeks and by 8 kPa after six weeks [14]. Park et al. performed only tongue-pressing effortful swallow (TPES) and had greater results, as the pressure was increased by approximately 8 kPa after four weeks [33]. Lazarus et al., on the other hand, found no significant difference in the mean tongue strength measures pre-post training. The patients however were instructed to perform the exercises at home alone. The authors found that the patient’s compliance with the exercise protocols was relatively poor [18].

Following our treatment protocol, the patients in the TG showed an anterior tongue pressure increase of 10.9 kPa after four weeks and by 15.7 kPa after six weeks. The posterior tongue pressure increased by 12.3 kPa after four weeks and by 17 kPa after six weeks. The highest increase was observed in the first two weeks of treatment. Improvement of the MIPs continued until the end of the eighth week.

Kraaijenga S. et al. observed that muscle strength was also increased in the first weeks of treatment in head and neck cancer individuals with chronic dysphagia [34]. Robbins et al. also found that stroke patients showed more than 60% of their overall improvement for both anterior and posterior sites after four weeks of exercise [15].

These findings demonstrate that high-intensity tongue-strengthening exercises can improve tongue pressures in patients after glossectomy. Since the pressure increase of the TG is approximately 5.6 times faster than that of the FUG, it is strongly advised for patients after glossectomy to follow an effective treatment protocol consistently.

### 4.2. Tongue Endurance

This is the first study that reports tongue endurance in both the anterior and posterior portion of the tongue in patients after glossectomy. Our findings suggest that the endurance of both regions increased significantly after the treatment. The most considerable effect of the training was at the end of the second week, similar to the increase in the MIPs at that period. The follow-up group also showed a statistically significant increase in tongue endurance compared to the baseline. However, the improvement was considerably lower compared to the TG.

Surprisingly, tongue endurance measurements were higher in the TG post-treatment compared to the RG. We must take into account that, as the TG has lower MIPs than the RG, the endurance task of the TG is performed at lower pressure levels than the RG. Lower pressure levels are known to result in longer endurance times [35]. Our findings agree with Lazarus et al., who also found that endurance is higher in head and neck cancer patients than healthy participants [23].

### 4.3. Swallowing Pressures

In the literature, in patients with oral cancer, tongue strength was measured as a separate task, rather than during the swallows. This is the first study that evaluates and records swallowing pressures over time pre-post treatment. Our results showed that the patient’s swallowing pressures were very low at baseline. Authors in the past found that healthy older individuals with reduced maximum isometric pressures tend to have lower lingual swallowing pressures as well [23].

Patients in our study in the TG significantly increased their swallow pressures post-treatment, which can be attributed to the increase in lingual pressure due to tongue-strengthening and accuracy exercises and dynamic swallow exercises. Training both the anterior and posterior tongue increases tongue pressure in the anterior and posterior portions of the tongue during swallowing [36]. A greater increase, however, appeared at the posterior portion of the tongue for both normal and forceful swallowing compared to the anterior. The posterior tongue provides the major propulsive force for transferring food and liquid from the oral cavity into the pharynx (8) and has been found to generate higher pressures during swallows of liquid and semisolid boluses [14].

The FUG also increased their swallowing pressures. The improvement, however, was much smaller and not statistically significant. As patients in the follow-up group did not exercise in strength and swallow tasks, swallow pressures did not significantly alter.

### 4.4. EAT-10, PAS, PSR, and MD

We found that patients in TG revealed high scores of residues in the pharyngeal wall and vallecular in both thick and solid boluses at baseline. Our data suggest that increased tongue-driving force post-treatment contributes to effective bolus clearance from the pharynx, minimizing the residues. Robbins et al. observed that increase in the lingual strength results in a longer pharyngeal response, possibly related to the reduction of the pharyngeal residue [15].

Swallowing exercises also contribute to the improvement of the swallowing function. Effortful swallow produces significantly greater muscle activity, generates greater pharyngeal pressure, and improves pharyngeal bolus clearance [37]. Mendelson maneuver reduces aspiration and improves the laryngeal range of motion, timing, and coordination of the pharyngeal swallow [38]. Supraglottic swallow prolongs airway closure duration and minimizes aspiration, especially in HNC patients [3].

The ability to eat most diet types (as solid boluses or thin liquids) decreased immediately after the tongue resection and then increased over time. As maximum tongue strength, endurance, and swallowing pressures improved in the TG, fewer patients received primary nutrition via PEG, and more patients received solid boluses. These findings correspond to the decreased score at EAT-10 < 3 post-treatment. It has been found that feeding status is strongly correlated to the EAT-10 score, the PAS scale, and anterior tongue strength [39].

Five patients were feeding orally, additionally to the PEG in the eighth week. Two of them had collapsed at the end of the sixth week and three of them did not continue the treatment after the end of the eighth week. Thus, we do not have data regarding the progress afterwards or if they needed more sessions for further improvement. The relationship between the pressures, the endurance, and the ability to handle some types of oral diet in patients after glossectomy needs further investigation in a larger number of patients.

Reduced tongue strength and low saliva swallow have been associated with longer meal durations and reduced food consumption in long-term care residents [12,40]. We found that mealtime duration decreased significantly post-treatment and was associated with the increase in the tongue and swallow pressures. To our knowledge, this is the first recording of the time needed for meal consumption in patients after tongue resection.

### 4.5. Limitations

We did not include and analyze parameters regarding the stage of the tumor pre-treatment, the extent of surgical resection, the tongue reconstruction, and the tissue defect post-surgically. The time from the surgery was 3–4 months for most patients, but for four patients it was 2 and 10 months. Finally, the measurement of the mealtime duration was based on patient reporting, and in the future should be in the presence of the trainer, with the same amount and consistency of food for each patient.

## 5. Conclusions

The high-intensity tongue strengthening exercise protocol provides quicker and greater improvement in tongue pressures and swallowing function than natural improvement.

## Figures and Tables

**Table 1:** Baseline characteristics of the participant groups.

	Therapy Group (TG)	Follow-Up Group (FUG)	Reference Group (RG)
N Males Females	17 7 (mean age = 54.5) 10 (mean age = 53.6)	8 4 (mean age = 52) 4 (mean age = 64.5)	31 17 (mean age = 51.7) 14 (mean age = 43.7)
Tumor location			n/a *
Lateral side	8	7	
Base of the tongue	9	1	
Adjuvant treatment			n/a *
Chemotherapy	5	4	
Chemo/radiotherapy	12	4	
Feeding status			All on full oral feeding
Oral solid	0		
Oral soft	10		
Partial oral feeding	0	8	
Non oral feeding	7		
Post-operative time			n/a *
2 months	2	1	
3 months	8	4	
4 months	6	3	
10 months	1		

* n/a: not applicable.

**Table 2:** Oropharyngeal swallowing measures pre-post treatment in the Therapy Group.

Therapy Group	Mean	Std. Deviation	*p*-Value
ΕAΤ10 total pre	28.00	8.81	0.000 *
ΕAΤ10 total post	2.93	0.96	
PAS liquids pre	4.86	2.35	0.971
PAS liquids post	4.71	13.05	
PAS thick pre	3.14	2.14	0.002 *
PAS thick post	1.07	0.27	
PAS solid pre	2.80	1.48	0.004 *
PAS solid post	1.00	0.00	
PSR pre thick pre	1.57	0.51	0.000 *
PSR post thick post	0.21	0.43	
PSR solid pre	1.80	0.42	0.000 *
PSR solid post	0.60	0.52	
MD pre (min)	43	3.50	0.000 *
MD post (min)	14	0.67	

Abbreviations: EAT-10, Eating Assessment Tool Questionnaire; PAS, Penetration-Aspiration Scale; PSR, Post-Swallow Residues; MD, Mealtime Duration * *p* < 0.05; Wilcoxon Paired Samples Test.

## Data Availability

The dataset analyzed in the current study is not publicly available. The anonymized data are available from the authors upon reasonable request.
